# Coexistence of *JAK2* and *CALR* mutations and their clinical implications in patients with essential thrombocythemia

**DOI:** 10.18632/oncotarget.10958

**Published:** 2016-07-30

**Authors:** Min-Gu Kang, Hyun-Woo Choi, Jun Hyung Lee, Yong Jun Choi, Hyun-Jung Choi, Jong-Hee Shin, Soon-Pal Suh, Michael Szardenings, Hye-Ran Kim, Myung-Geun Shin

**Affiliations:** ^1^ Departments of Laboratory Medicine, Chonnam National University Medical School and Chonnam National University Hwasun Hospital, 322 Seoyang-ro, Hwasun-eup, Hwasun-gun, Jeollanam-do, South Korea; ^2^ Brain Korea 21 Plus Project, Chonnam National University Medical School, Gwangju, South Korea; ^3^ Environmental Health Center for Childhood Leukemia and Cancer, Chonnam National University Medical School and Chonnam National University Hwasun Hospital, Hwasun-eup, Hwasun-gun, Jeollanam-do, South Korea; ^4^ Department of Cell Therapy, Fraunhofer Institute for Cell Therapy and Immunology, Leipzig, Germany; ^5^ College of Korean Medicine, Dongshin University, Naju, Jeollanam-do, South Korea

**Keywords:** calreticulin, coexistence, essential thrombocythemia, Janus kinase 2, myeloproliferative disorders

## Abstract

Janus kinase 2 (*JAK2*) and calreticulin (*CALR*) constitute the two most frequent mutations in essential thrombocythemia (ET), and both are reported to be mutually exclusive. Hence, we examined a cohort of 123 myeloproliferative neoplasm (MPN) patients without *BCR-ABL1* rearrangement and additional ET patients (n=96) for coexistence of *JAK2* and *CALR* mutations. The frequency of *CALR* mutations was 20.3% in 123 MPN patients; 31.1% in ET (n=74), 25% in primary myelofibrosis (n=4) and 2.2% in polycythemia vera (n=45). *JAK2* and *CALR* mutations coexisted in 7 (4.2%) of 167 ET patients. Clinical characteristics, progression-free survival (PFS), and elapsed time to achieve partial remission across 4 groups (*JAK2+/CALR+, JAK2+/CALR-, JAK2-/CALR+, JAK2-/CALR-*) were reviewed. The *JAK2+/CALR-* group had higher leukocyte counts and hemoglobin levels and more frequent thrombotic events than *JAK2-/CALR-* group. *JAK2* mutations have a greater effect on the disease phenotype and the clinical features of MPN patients rather than do *CALR* mutation. *JAK2+* groups showed a tendency of poor PFS than *JAK2*- groups regardless of *CALR* mutation. *CALR+* was a predictor of late response to the treatment. Our study also showed that thrombosis was more frequent in ET patients with type 2 *CALR* mutations than in those with type 1 *CALR* mutations.

## INTRODUCTION

Myeloproliferative neoplasms (MPNs) are clonal diseases of hematopoietic stem cells. MPNs usually exhibit terminal myeloid cell expansion in the peripheral blood [1]. *BCR-ABL1* rearrangement-negative MPNs can be classified as polycythemia vera (PV), essential thrombocythemia (ET), or primary myelofibrosis (PMF). In general, 90–95% of PV patients and 50–60% of ET and PMF patients have Janus kinase 2 (*JAK2)* V617F mutations, and 3% of PV patients have *JAK2* exon 12 mutations [2, 3]. The discovery of *JAK2* mutations has been a great help in the diagnosis of *BCR-ABL1* rearrangement-negative MPNs.

Subsequent studies identified a thrombopoietin receptor (*MPL*) exon 10 mutation in 3–5% of ET patients and 5–8% of PMF patients without *JAK2* mutations [4–6]. In 2008, the World Health Organization (WHO) designated *JAK2* and *MPL* mutations as MPN diagnostic criteria [7, 8]. Recently, mutations in the calreticulin (*CALR*) gene, which encodes the calreticulin protein, were found in 50–80% of ET and PMF patients, all of whom lacked *JAK2* and *MPL* mutations [9, 10]. The discovery of *CALR* mutations further assists the diagnosis of *BCR-ABL1* rearrangement-negative MPNs, and it is thought that *CALR* mutations will eventually be included in the ET and PMF diagnostic criteria of the WHO [11].

More than 50 types of *CALR* mutations have been reported, and more than 80% of *CALR* mutations are characterized by a 52-bp loss (p.L367fs*46) or a 5-bp TTGTC insertion (p.K385fs*47) [12]. A previous study detected *CALR* mutations in 67% of ET patients and 88% of PMF patients with *JAK2*/*MPL* mutation-negative MPNs. In their study, the clinical features of patients with *CALR* mutations, compared with those with *JAK2* mutations, were as follows; lower levels of hemoglobin and white blood cells, lower risk of thrombosis, higher platelet counts, and better survival rates [9]. Another study also found *CALR* mutations in patients with *JAK2/MPL* mutation-negative MPNs [10]. In that study, positive rates of *CALR* mutation were 71%, 56%, and 86% in ET, PMF, and post-ET myelofibrosis patients, respectively, and *CALR* mutations were associated with higher platelet counts, lower hemoglobin levels, and more extensive fibrosis progression.

Most studies of *CALR* mutations were performed on *JAK2* mutation-negative MPN patients, and at present, there is little information about the coexistence of the *JAK2* V617F mutation and *CALR* exon 9 mutations [13–18]. A systematic investigation of patients with both mutations and of their combined effects on the pathophysiology, treatment outcome, phenotype, and clinical features of patients with MPNs is needed [12]. Some current studies report lower *CALR* mutation frequencies than those reported in earlier studies, and *CALR* mutation frequencies are thought to differ according to race and country [9, 14, 19, 20]. Patients with type 1 and type 2 *CALR* mutations have different prognoses, and previously it was reported that MPN patients with type 1 *CALR* mutation but not type 2 *CALR* mutations had a better prognosis than those with *JAK2* mutations [21].

The purpose of our study was to determine the frequency of *CALR* mutations (first cohort of 123 MPN patients) and coexisting *CALR* and *JAK2* mutations (combined cohort subset of 167 ET patients) in Korean patients with *BCR-ABL1* rearrangement-negative MPNs. We also examined the clinical features, progression-free survival, and treatment response of ET patients with these mutations.

## RESULTS

### *CALR* mutation frequency in patients with *BCR-ABL1* rearrangement-negative MPNs

**Figure 1:**
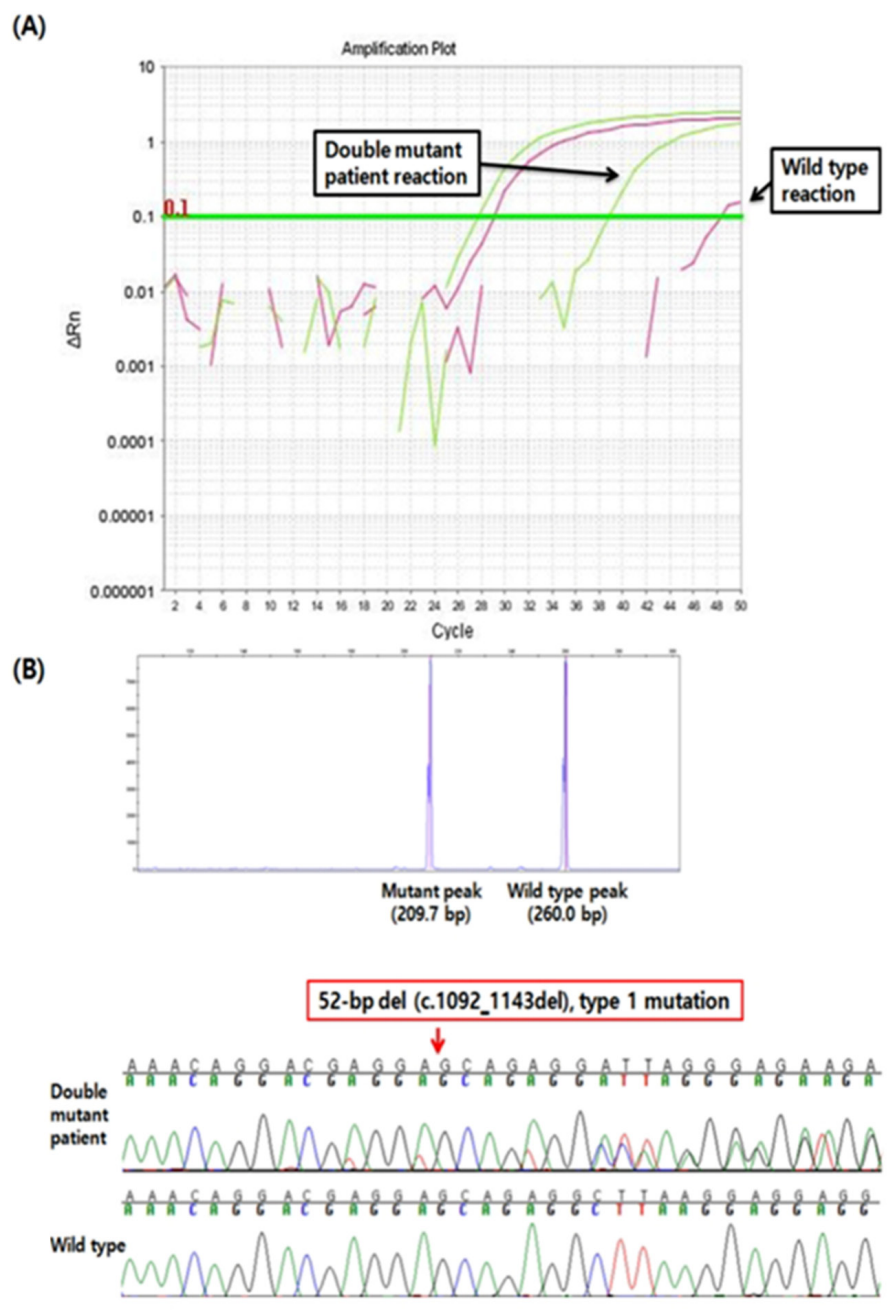
Representative results of *JAK2* and *CALR* mutations in a patient with both mutations **A.** Real-time quantitative polymerase chain reaction amplification plot of a *JAK2* V617F mutation shows the amplification curves for the wild-type (marked in purple) and the mutant (marked in green) alleles. The wild-type control had a C_T_ of 48, and the double mutation had a C_T_ of 39. **B.** *CALR* exon 9 fragment analysis sizing plot shows an abnormal peak with about a 50-bp difference compared with the wild-type peak (top). The results of direct sequencing show a type 1 mutation resulting from a 52-bp deletion (c.1092_1143del) (bottom).

*JAK2* V617F, *JAK2* exon 12, *MPL* exon 10, and *CALR* exon 9 mutation frequencies in first cohort of 123 patients with *BCR-ABL1* rearrangement-negative MPNs are shown in Table 1. The overall mutation frequencies for *CALR* exon 9 mutations were 20.3% (25/123) in all MPN patients, 31.1% (23/74) in ET patients, 25.0% (1/4) in PMF patients, and 2.2% (1/45) in PV patients. Five of the 26 ET patients with a *JAK2* V617F mutation also had a *CALR* exon 9 mutation (Figure 1). The sample from 1 of above 5 patients contained a fragment whose size differed from that of the normal *CALR* exon 9 fragment as determined via DNA fragment analysis (the screening test for *CALR* mutations). However, this fragment did not contain any *CALR* mutations as determined via direct sequencing (the second test). A *CALR* exon 9 mutation was also observed in a PV patient who was negative for *JAK2* mutations; however, this mutation was only observed in the fragment analysis and not via direct sequencing. Above cases with abnormal size of *CALR* exon 9 fragment were considered to be mutated (Table 1).

**Table 1:** The mutational status of 123 patients with *BCR-ABL1* rearrangement-negative myeloproliferative neoplasms

Mutation	ET, n (%) (n = 74)		PMF, n (%) (n = 4)		PV, n (%)(n = 45)		Total, n (%)	
	Mutant	Wild type	Mutant	Wild type	Mutant	Wild type	Mutant	Wild type
*JAK2* V617F	26(35.1)	48(64.9)	1(25.0)	3(75.0)	19(42.2)	26(57.8)	46(37.4)	77(62.6)
JAK2 exon 12	N/T	N/T	N/T	N/T	0	26	0	26
MPL exon 10	1(2.1)	47(97.9)	2(66.7)	1(33.3)	N/T	N/T	3(5.9)	48(94.1)
CALR exon 9	23*(31.1)	51(68.9)	1(25.0)	3(75.0)	1*(2.2)	44(97.8)	25(20.3)	98(79.7)

ET, essential thrombocythemia; PMF, primary myelofibrosis; PV, polycythemia vera; CNL, chronic neutrophilic leukemia; n, number; N/T, not tested.

* Two patients with ET and 1 patient with PV had a low mutant allele burden; therefore, mutations were detected via fragment analysis rather than direct sequencing. Those cases were considered to be mutated.

### *JAK2* and *CALR* mutation frequency in ET patients

Among the 74 ET patients out of 123 MPN patients, 1 had an *MPL* exon 10 mutation and 2 did not have a *CALR* exon 9 mutation detectable via direct sequencing; these 3 patients were excluded from our analysis. Accordingly, the samples from 71 ET patients (out of the 123 patients) and an additional 96 ET patients were examined for *JAK2* and *CALR* mutation. In total, combined cohort subset of 167 ET patients were classified as follows: *JAK2*+/*CALR*+, *JAK2*+/*CALR*-, *JAK2*-/*CALR+*, and *JAK2*-/*CALR*-. The frequency of each subgroup of ET patients were as follows: 4% (n=7) were *JAK2+/CALR+*, 62% (n=103) were *JAK2+/CALR-*, 11% (n=19) were *JAK2-/CALR+*, and 23% (n=38) were *JAK2-/CALR-* (Table 2).

**Table 2:** Clinical and laboratory characteristics of 167 patients with essential thrombocythemia according to the mutational status of *JAK2* and *CALR*

Characteristic	*JAK2*+/*CALR*+ (A)	*JAK2*+/*CALR*- (B)	*JAK2*-/*CALR*+ (C)^‡^	*JAK2*-/*CALR*- (D) ^‡^	*P* value					
					AvsB	AvsC	AvsD	BvsC	BvsD	CvsD
Patients, n (%)	7 (4)	103 (62)	19 (11)	38 (23)	-	-	-	-	-	-
Male, n^†^	3	53	10	13	0.714	1.000	0.686	1.000	0.087	0.253
Age at onset, years, SE (med; ran)^*^	7.0(62;29-77)	1.2(63;6-87)	3.3(62;25-80)	2.9(52;11-80)	0.783	0.977	0.511	0.619	0.008	0.152
WBC count, × 10^9^/L, SE(med; ran)^*^	1.5(8.4;3.6–16.9)	0.7(12.7;5.1–59.6)	0.8(9.5;4.5–18.8)	0.5(9.0;4.4–18.3)	0.006	0.355	0.594	0.002	<0.0001	0.286
Hemoglobin, g/dL, SE(med; ran)^*^	0.83(13.4;9.6–15.1)	0.20(13.9;8.4–19.5)	0.33(13.5;10.1–15.9)	0.32(12.3;6.6–17.1)	0.225	0.685	0.707	0.188	0.0003	0.080
Platelet count, × 10^9^/L, SE(med; ran)^*^	110(1006;471-1186)	24(933;487-1935)	97(1021;557-2232)	50(998;456-1648)	0.995	0.355	0.719	0.089	0.566	0.306
Lactate dehydrogenase, mU/mL, SE(med; ran)^*^	88(486;185–969)	19(557;192–1157)	48(481;200–1000)	31(462;195–932)	0.233	0.885	0.835	0.272	0.026	0.478
Splenomegaly, n (%)^†^	3 (43)	35 (34)	7 (37)	4 (11)	0.691	1.000	0.064	0.799	0.006	0.031
Thrombotic events, n (%)^†^	0	32 (31)	3 (16)	3 (8)	0.104	0.540	1.000	0.270	0.004	0.389
Plateletpheresis, n (%)^†^	4 (57)	34 (33)	8 (42)	12 (32)	0.232	0.665	0.225	0.444	1.000	0.558
Transformation to secondary MF or AL, n (%)^†^	0	7 (7)	0	0	1.000	N/A	N/A	0.594	0.190	N/A

n, number; SE, standard error; med, median; ran, range; WBC, white blood cell; MF, myelofibrosis; AL, acute leukemia; N/A, not applicable.

* *P* values were calculated using the Mann-Whitney *U* test.

† *P* values were calculated using the Fisher's exact test.

‡ Group C and D were all negative for *MPL* exon 10 mutation.

### Clinical features of the ET patient groups classified according to *JAK2* and *CALR* mutations

There were no significant differences between the *JAK2*+/*CALR*+ group and the other groups except that white blood cell counts were lower in the *JAK2*+/*CALR*+ group than in the *JAK2*+/*CALR-* group. The *JAK2*+/*CALR*- group had higher white blood cell counts and levels of hemoglobin and lactate dehydrogenase and higher rates of splenomegaly and thrombosis than did the *JAK2*-/*CALR*-group. Also, the *JAK2*+/*CALR*- group contained more elderly patients than did the *JAK2*-/*CALR*-group. Meanwhile, the *JAK2-*/*CALR+* group had higher rates of splenomegaly than did the *JAK2-*/*CALR*- group.

**Figure 2:**
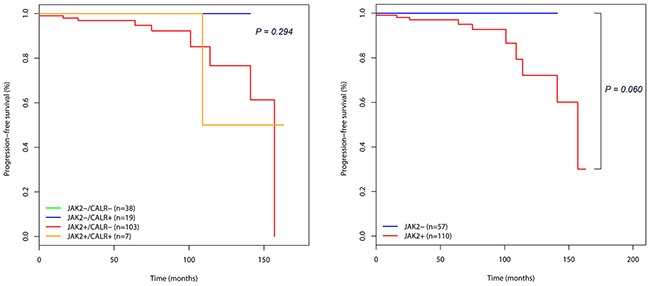
Kaplan-Meier survival curves showing the progression-free survival (PFS) in 167 essential thrombocythemia (ET) patients according to mutational status **A.** PFS categorized according to the mutational status of *JAK2* and *CALR*. The survival line of *JAK2-/CALR-* group (green) was hidden in the back of that of *JAK2-/CALR+* group (blue). **B.** PFS categorized according to the mutational status of *JAK2*.

In *JAK2-* groups (*JAK2*-/*CALR*+ and *JAK2*-/*CALR*-), no patient had experienced disease progression. Therefore, *JAK2-* groups showed better PFS than *JAK2+* groups (*JAK2*+/*CALR*+ and *JAK2*+/*CALR*-), albeit without statistical significance (Table 3 and Figure 2B, *P*=0.060). Then again, we performed multivariable Cox regression analyses, examining age, sex, white blood cell count, hemoglobin, platelet count, lactate dehydrogenase, mutational status of *JAK2*/*CALR*, splenomegaly, and thrombosis. As a result, age (HR = 1.079; 95 % CI, 1.008–1.155; *P* = 0.029), lactate dehydrogenase (HR = 1.003; 95 % CI, 1.000–1.006; *P* = 0.030), thrombosis (HR = 14.512; 95 % CI, 2.991–70.410; *P* = 0.001) remained an independent predictor for PFS.

**Table 3:** Disease progression of 167 patients with essential thrombocythemia according to the mutational status of *JAK2* and *CALR*

Characteristics	*JAK2*+/*CALR*+ (A)	*JAK2*+/*CALR*- (B)	*JAK2*-/*CALR*+ (C)	*JAK2*-/*CALR*- (D)	*P* value^*^					
					AvsB	AvsC	AvsD	BvsC	BvsD	CvsD
Disease progression (%) in 4 groups	1/7(14.3)	9/103(8.7)	0/19(0.0)	0/38(0.0)	0.708	0.157	0.480	0.171	0.217	N/A
Disease progression (%) in 2 groups (*JAK2*+ vs *JAK2*-)	10/110(9.1)		0/57(0.0)		0.060					

N/A, not applicable.

* *P* values were calculated using the Mann-Whitney *U* test.

**Figure 3:**
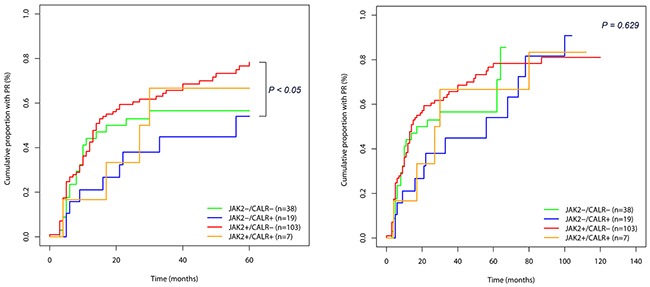
Treatment response (cumulative proportion to achieve partial remission) in 167 essential thrombocythemia (ET) patients according to mutational status **A.** Cumulative proportion with PR categorized according to the mutational status of *JAK2* and *CALR* when follow-up duration was 5 years. **B.** Cumulative proportion with PR categorized according to the mutational status of *JAK2* and *CALR* when follow-up duration was 10 years.

In the treatment response, the 5-year cumulative proportion with partial remission was best in *JAK2+/CALR-* group as 78.3% and the next was *JAK2+/CALR+, JAK2-/CALR-, JAK2-/CALR+* in orders (Table 4 and Figure 3A). Especially, the 5-year cumulative proportion with partial remission in *JAK2+/CALR-* group (78.3%) was higher than that of *JAK2-/CALR+* group (54.1%) with statistical significance (Table 4, *P<*0.05). The 10-year cumulative proportion with partial remission were not statistically different between the 4 groups (Table 4 and Figure 3B). Meanwhile, the elapsed time to achieve partial remission of half of patients in each group was shortest in *JAK2+/CALR-* as 14 months and the next was *JAK2-/CALR-, JAK2+/CALR+, JAK2-/CALR+* in orders. The elapsed time to achieve partial remission in half of patients (PR_50_) of *JAK2-/CALR+* group was 56 months, it was 4 times longer than that of *JAK2+/CALR-* group (Table 5).

**Table 4:** Treatment response^*^ of 167 patients with essential thrombocythemia according to the mutational status of *JAK2* and *CALR*

Follow-up duration	*JAK2*+/*CALR*+ (A)	*JAK2*+/*CALR*- (B)	*JAK2*-/*CALR*+ (C)	*JAK2*-/*CALR*- (D)	*P* value^†^					
					AvsB	AvsC	AvsD	BvsC	BvsD	CvsD
5 years	66.7^*^(0.00–89.2)	78.3(66.6–85.9)	54.1(19.8–73.7)	56.6(35.9–70.6)	0.450	0.554	0.997	0.043	0.219	0.395
10 years	83.3(0.3–97.2)	81.0(68.6–88.6)	90.8(42.2–98.5)	85.5(24.8–97.2)	0.668	0.978	0.640	0.236	0.416	0.183

* The cumulative proportion to achieve partial remission (%) and its 95% confidential interval.

† *P* values were calculated using the Mann-Whitney *U* test

**Table 5:** Elapsed time to achieve partial remission in half of patients (PR_50_) according to the mutational status of *JAK2* and *CALR*

Characteristics	*JAK2*+/*CALR*+ (A)	*JAK2*+/*CALR*- (B)	*JAK2*-/*CALR*+ (C)	*JAK2*-/*CALR*- (D)
Elapsed time to PR_50_, months (95% CI)	28.5 (17–N/A)	14 (12–27)	56 (21–N/A)	20 (10–N/A)

CI, confidence interval; N/A, not applicable

### *CALR* mutation type analysis in ET patients

Twenty-six of the 167 ET patients had *CALR* mutations. These mutations were as follows: a 52-bp loss (type 1, pL367fs*46; 13patients), a 5-bp insertion (type 2, p.K385fs*47; 7 patients), and c.1095_1140del (type 3; 1 patient) (Table 6). The remaining 5 patients had novel mutations including c.1105_1138del (p.E369fs*50), c.1103_1136del (p.K368fs*51), c.1093_1126del (p.Q365fs*54), c.1144del (p.D382fs*48), and c.1132_1154 delins TGTC (p.E378fs*46), which had both a deletion and an insertion.

**Table 6:** Types and frequencies of *CALR* mutation in 167 patients with essential thrombocythemia

*CALR* mutation^*^	cDNA annotation	C-terminal amino acid sequence^†^	Protein annotation	Frequency	
				n	%
Type 1	c.1092_1143del	AAEKQMKDKQDEEQ RTRRMM**RTKMRMR** **RMRRTRRKMRRKMSPARP** **RTSCREACLQGWTEA**-	p.L367fs*46	13	50.0
Type 2	c.1154_1155 insTTGTC	AAEKQMKDKQDEEQRLKEE EEDKKRKEEEEAEDIVR RMMRTKM **RMRRMRRTRR** **KMRRKMSPARP** **RTSCREACLQGWTEA**-	p.K385fs*47	7	26.9
Type 3	c.1095_1140del	AAEKQMKDKQDE EQRQRTRRMMRT KMRM**RRMRRT** **RRKMRRKMSPARP** **RTSCREACLQGWTEA**-	p.L367fs*48	1	3.8
Novel	c.1105_1138del	AAEKQMKDK QDEEQRLKRRQRTR RMMRTKM**RMRRMR** **RTRRKMRRKMSPARPRTS** **CREACLQGWTEA**-	p.E369fs*50	1	3.8
Novel	c.1103_1136del	AAEKQMKDK QDEEQRLRRRQRTRR MMRTKM**RMRRMRRT** **RRKMRRKMSPARPR** **TSCREACLQGWTEA**-	p.K368fs*51	1	3.8
Novel	c.1093_1126del	AAEKQMKDKQD EEAKRRRRQRTRR MMRTKM**RMRRM** **RRTRRKMRRKMSPARPR** **TSCREACLQGWTEA**-	p.Q365fs*54	1	3.8
Novel	c.1132_1154 delinsTGTC	AAEKQMKDKQDEEQRLKEEEED KKRKCRRMMRTKM **RMRRMRRTRRKMRR** **KMSPARPRT** **SCREACLQGWTEA**-	p.E378fs*46	1	3.8
Novel	c.1144del	AAEKQMKDKQD EEQRLKEEEEDKKRKEEEEQ RTRRMMRTKM**RMRRMRRTRRKMR** **RKMSPARPRTSCREACLQGWTEA**-	p.D382fs*48	1	3.8
Total				26	100
Wild type reference sequence		AAEKQMKDKQD EEQRLKEEEEDKKR KEEEEAEDKEDDE DKDEDEEDEEDKEED EEEDVPGQA**KDEL**-			

* “Novel” refers to mutation types not described in the literature [1–3].

† Amino acids in bold show the novel C-terminal peptide sequence that commonly results from an altered reading frame lacking the KDEL (endoplasmic reticulum retention signal) motif.

### Clinical and laboratory characteristics of ET patients with type 1 and type 2 *CALR* mutations

The characteristics of ET patients with type 1 *CALR* mutations (n=9) and type 2 *CALR* mutations (n=6) were compared; neither group had a *JAK2* mutation (Table 7). Thrombosis was more frequent in the type 2 group than the type 1 group. There were no significant differences in age, sex, hematologic parameters, or frequency of splenomegaly or therapeutic plateletpheresis between the groups. The PFS rates of the 2 groups were not significantly different (*P*=1.000).

**Table 7:** Clinical and laboratory characteristics of ET patients with type 1 or type 2 *CALR* mutations

Characteristic	Type 1 (n = 9)	Type 2 (n = 6)	P
Male, n^†^	4	3	1.000
Age at onset, years, median (range)^*^	62 (49–76)	64 (25–80)	0.864
WBC count, × 10^9^/L, median (range)^*^	7.7 (4.5–15.8)	11.1 (6.2–13.6)	0.224
Hemoglobin, g/dL, median (range)^*^	13.3 (10.1–15.5)	13.2 (11.5–15.9)	1.000
Platelet count, × 10^9^/L, median (range)^*^	1021 (557–1839)	1074 (712–2232)	0.864
Lactate dehydrogenase, mU/mL, median (range)^*^	491 (267-872)	467 (200–1000)	0.864
Splenomegaly, n (%)^†^	3 (33.3%)	2 (33.3%)	1.000
Thrombotic events, n (%)^†^	0	3 (50.0%)	0.044^‡^
Plateletpheresis, n (%)^†^	3 (33.3%)	4 (66.7%)	0.315
Transformation to secondary MF or AL, n^†^	0	0	N/A

ET, essential thrombocythemia; n, number; WBC, white blood cell; MF, myelofibrosis; AL, acute leukemia; N/A, not applicable.

* *P* values were calculated using the Mann-Whitney *U* test.

† *P* values were calculated using Fisher's exact test.

‡ Statistical significance

## DISCUSSION

*CALR* encodes a multi-functional Ca^2+^-binding protein termed calreticulin that is primarily found in the nucleus, cell membrane, and extracellular matrix [22]. The *CALR* gene is located on chromosome 19p13.2, is composed of 9 exons, and is about 4.2 kb [23, 24]. Calreticulins participates in Ca^2+^ homeostasis, the removal of mis-folded proteins, cell adhesion, immune responses to cancers, phagocytosis, and signaling [22, 25]. Unusual manifestations of calreticulin have been observed in various cancers occurred in ovary, pancreas, and breast [26–28] and point mutations in the *CALR* gene promoter have been reported in patients with schizophrenia [29].

Previously, the frequencies of *CALR* exon 9 mutations in MPN patients without *JAK2* or *MPL* mutation were reported to 67% in ET patients and 88% in PMF patients in one study [9], and 82% in ET patients and 80% in PMF patients in other study [10]. In the first cohort of 123 MPN patients of this study, the frequency of *CALR* mutations was 31.1% in all ET patients and 25.0% in all PMF patients, regardless of the *JAK2* or *MPL* mutation (Table 1). These rates of *CALR* mutation seems to be diverse according to race and country; previously reported rates of *CALR* mutations for ET patients are 67.0% (considering ET patients without *JAK2* or *MPL* mutation) in Austria [9], 15.5% (considering all ET patients) in Italy [19], 22.5% (considering all ET patients) in Taiwan [14], and 25.0% (considering all ET patients) in Han China [20]. In addition to ET and PMF patients, *CALR* mutations have also been observed in 3(13.0%) of 24 patients with refractory anemia with ringed sideroblasts and marked thrombocytosis; none of the 3 patients had *JAK2* or *MPL* mutations [9]. In other study, *CALR* mutations have also been detected in patients with myelodysplastic syndrome (8.3%; n=120), chronic myelomonocytic leukemia (3%; n=33), and atypical chronic myelogenous leukemia (CML) (3.4%; n=29) [10].

In this study, a small number of mutant alleles were observed in one PV patient in a *CALR* DNA fragment analysis. This patient was a 64-year-old man with a white blood cell count of 7.5 × 10^9^/L, a hemoglobin level of 19.4 g/dL, a hematocrit level of 54.5%, a platelet count of 291 × 10^9^/L, and a normal erythropoietin level. He did not have any *JAK2* V617F or *JAK2* exon 12 mutations. Although an earlier study did not detect any *CALR* mutations in PV patients, a more recent study found *CALR* mutations in 2 PV patients negative for *JAK2* mutations and demonstrated the existence of clones of peripheral blood granulocyte and burst-forming unit-erythroid progenitors [13]. It also suggested the possibility of a relationship between *CALR* mutations and the PV phenotype, justifying recommendations for *CALR* mutation tests in PV patients negative for *JAK2* mutations. Because *CALR* mutations occur in multipotent progenitor cells that can differentiate into erythroid and myeloid lineage cells [10], we hypothesize that these mutations play a role in tumorigenesis not only in the megakaryocytes of ET and PMF patients, but also in the erythroid cells of PV patients.

Allele-specific PCR (AS-PCR), DNA fragment analysis, direct sequencing, and immunostaining are commonly used to detect *CALR* mutations [9, 30, 31]. Chi *et al*. previously compared AS-PCR with DNA fragment analysis using capillary gel electrophoresis. In their report, a very small number of mutant alleles that could not be detected via agarose gel electrophoresis were detectable via fragment analysis, which had a sensitivity of less than 5% [30]. In this study, samples from two ET patients and one PV patient contained fragments with atypical sizes in fragment analysis; however, these variant alleles were not detectable via direct sequencing. The percentages of mutant alleles detected via fragment analysis were 5.1% and 4.8% in two ET patients, respectively, and 14.6% in the PV patient. The percentage of mutant alleles in total DNA required for detection via direct sequencing is usually more than 10–15%. Accordingly, mutations might not have been detected in the patients in our study with percentages less than 15%.

A previous domestic study showed that 27.4% of ET patients (n=84) and 22.4% of PMF patients (n=49) had *CALR* mutations; however, none of these patients also had *JAK2* mutations [32]. In that study, *CALR* mutations were examined via direct sequencing, whereas *JAK2* mutations were examined via AS-PCR. Detection of *JAK2* V617F mutations via AS-PCR requires a sensitivity of about 3%, and real-time PCR has a sensitivity of less than 1% [33]. Although the *CALR* mutation frequencies in the ET and PMF patients in our study were similar to above study, the frequencies of simultaneous *JAK2* and *CALR* mutations were different, presumably owing to sensitivity differences between the detection methods. In particular, the allele burden of *CALR* mutations was very small in only one of the 8 patients of *JAK2*+/*CALR*+ group of this study, accordingly the *CALR* mutation was not detected via direct sequencing in the one patient. By contrast, the allele burden of *JAK2* V617F mutations was very small in all 8 patients of *JAK2*+/*CALR*+ group of this study (mean, 0.21%; range, 0.10–0.38) (Table 8). Therefore, it appears that detection sensitivity is much more critical for detecting *JAK2* mutations than for detecting *CALR* mutations. The amount of *JAK2* mutant alleles in the *JAK2*+/*CALR*+ patients in previous studies was also very small (below 5%, Table 8).

**Table 8:** Summary of patient characteristics, mutation types, and allele burdens in patients with both *JAK2* and *CALR* mutations in this study and previous studies

No. Case	Diagnosis	Sex	Age	*CALR* mutation	*CALR* allele burden (%)	*JAK2* allele burden (%)	Reference
1	ET	U	U	U	U	U	[4]
2	RARS-T	F	73	c.1129_1138del	U	4	[5]
3	PMF	U	U	c.1092_1143del (type 1)	U	U	[6]
4	ET	F	79	c.1094_1139del (type 6)	10.5	<1	[7]
5–8	PMF	U	U	U	U	U	[8]
9	ET	U	U	c.1092_1143del (type 1)	U	0.03	[9]
10	ET	F	62	c.1095_1140del (type 3)	24.22	0.16	this study
11	ET	M	29	c.1144del	50.37	0.27	this study
12	ET	F	71	c.1092_1143del (type 1)	61.08	0.12	this study
13	ET	F	61	U^*^	4.47	0.10	this study
14	ET	F	74	c.1092_1143del (type 1)	89.48	0.38	this study
15	ET	M	37	c.1092_1143del (type 1)	40.65	0.14	this study
16	ET	M	57	c.1154_1155insTTGTC (type 2)	36.92	0.26	this study
17	ET	F	77	c.1092_1143del (type 1)	47.54	0.28	this study

ET, essential thrombocythemia; RARS-T, refractory anemia with ringed sideroblasts and marked thrombocytosis; PMF, primary myelofibrosis; U, unknown; F, female; M, male.

* *CALR* mutations were detected only via gene fragment analysis owing to low allele burden.

In a previous study, ET patients with *CALR* mutations appeared to have lower hemoglobin levels and white blood cell counts, higher platelet counts, a lower risk of thrombosis, and a much higher rate of survival and fibrosis progression than ET patients with *JAK2* mutation [9, 10]. In this study, there were no significant differences in clinical parameters except white blood cell counts, between patients with *CALR* mutations and those with *JAK2* mutations. There were, however, significant differences between patients with *JAK2* mutations (rather than *CALR* mutations) and those in the *JAK2*-/*CALR*- group (Table 2). This finding suggests that *JAK2* mutations have a greater effect on the disease phenotype and clinical characteristics than do *CALR* mutations.

All types of *CALR* mutations reported thus far result in frameshifts that create a new C-terminal domain of the protein, which consists of at least 36 amino acids. In contrast to wild type in which amino acid with a wide range of negative charge is appeared, these new sequences make amino acid which is positively charged in many parts, resulting in a protein defective in endoplasmic reticulum signaling. This defect affects the intracellular localization, stability, and function of *CALR* and eventually it is related to carcinogenesis [9, 10].

In recent studies of ET patients, the percentages of type 1 and type 2 *CALR* mutations were 46% and 38%, respectively [34]. These percentages are similar to those reported here: 50% in type 1 and 27% in type 2. The results for PMF patients were more variable in previous studies. In a Western study, 80% of *CALR* mutations in PMF patients were type 1, and 11% were type 2 [21]. In contrast, 32% were type 1 and 34% were type 2 in a Chinese study [35], and 82% were type 1 and 9% were type 2 in a Korean study (n = 11) [32]. In this study, one (25%) of 4 PMF patients had a type 2 *CALR* mutation. The differences between studies may reflect the different sensitivities of the detection methods used or the different genetic backgrounds of PMF pathogenesis in the different ethnic groups [36].

In a study of 1,027 ET patients, both type 1 and type 2 *CALR* mutations were associated with higher platelet counts, lower white blood cell counts, and lower hemoglobin levels than *JAK2* mutations [37]. However, type 1 was more frequent in men, and type 2 was more frequent in younger individuals and correlated with significantly higher platelet counts. Therefore, it appears that the type of *CALR* mutation influences platelet formation differently.

Recently, a multicenter study compared the survival rate of *CALR*-mutated ET and *JAK2*-mutated ET patients [38]. The result showed a tendency that the survival rate of *CALR*-mutated ET is better than that of *JAK2*-mutated ET, but it was not statistically significant.

Current study showed the tendency that ET patients without mutated *JAK2* showed the better PFS than those with mutated *JAK2* regardless of *CALR* mutation (Figure 2B), but it was statistically not significant (*P*=0.060). Also we found that age, level of lactate dehydrogenase, and thrombotic event was an independent prognostic factor for a PFS outcome. ET patients with type 2 *CALR* mutations had a significantly higher frequency of thrombosis than those with type 1 mutations (Table 7), and although not significant, they tended to have a lower PFS rate (detailed data not shown).

Differently from PFS, the elapsed time to achieve the partial remission was significantly longer in *CALR-*mutated groups. This finding was previously not reported in any other studies and implies the possibility of refractoriness of *CALR* mutation to the current treatment regimens. More studies with larger patient cohorts and about the pathophysiology of *CALR* mutation are required to confirm the clinical impact of *CALR* mutation to the survival and treatment response.

In conclusion, we found that *CALR* mutations can coexist with *JAK2* mutations, in contrast to earlier reports. Coexistence, however, did not definitively affect prognosis or clinical features. Our data suggest that *JAK2* mutations have a greater effect on the disease phenotype and the clinical features of ET patients rather than do *CALR* mutation. Mutated *JAK2* group of ET patients showed inferior PFS regardless of *CALR* mutation, but they revealed early response to treatment than mutated *CALR* group. This study also showed that thrombosis was more frequent in ET patients with type 2 *CALR* mutations than in those with type 1 *CALR* mutations.

## METHODS

### Patients

**Figure 4:**
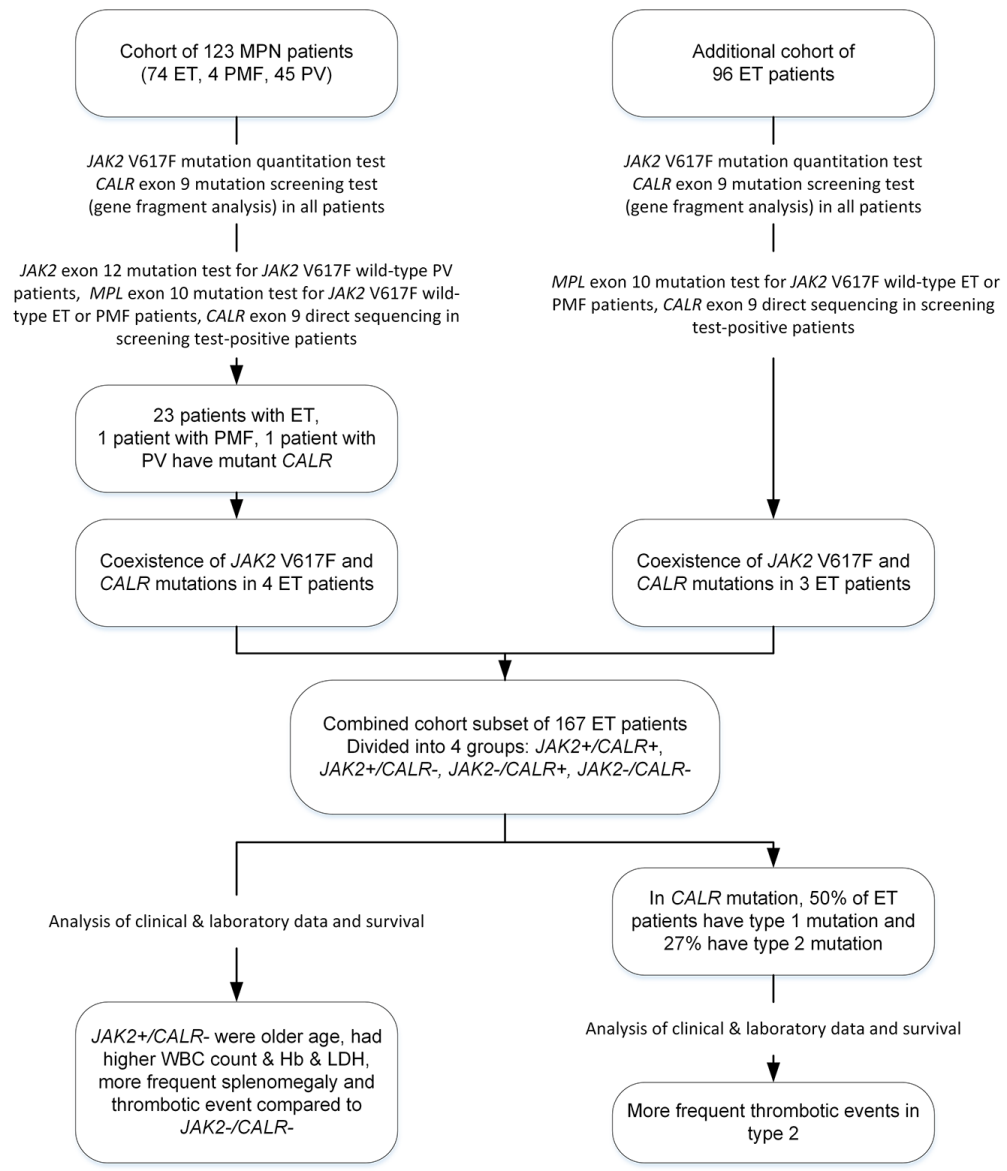
Flow chart describing the patient cohorts analyzed in this study A total of 219 patients with *BCR-ABL1* rearrangement-negative MPNs were enrolled. Because coexisting *JAK2* and *CALR* mutations were observed in the ET patients in the first patient cohort (n = 123), a second cohort of 96 ET patients was added. According to the mutational status of *JAK2* or *CALR* and the type of *CALR* mutation, the clinical and laboratory characteristics were analyzed in the 167 ET patients. Abbreviations: ET, essential thrombocythemia; PMF, primary myelofibrosis; PV, polycythemia vera; CNL, chronic neutrophilic leukemia; WBC, white blood cell, Hb, hemoglobin; LDH, lactate dehydrogenase.

The frequency of *CALR* mutations was determined in bone marrow (BM) samples from 123 Korean patients who were diagnosed with *BCR-ABL1* rearrangement-negative MPNs. Among these 123 MPN patients, 74 had ET, 4 had PMF, and 45 had PV. All diseases were diagnosed according to the criteria of the WHO [7, 39]. The clinical and diagnostic information of MPN patients was obtained from the electronic medical records of our hospital. Seventy-three (59.3%) out of the 123 patients were men and 50 were (40.7%) women, and the median age was 55 years (range, 4–84 years). We also examined BM samples from 96 ET patients, who were additionally included to current study cohort, to determine the frequency of coexisting *JAK2* and *CALR* mutations and their association with clinical features and prognosis (Figure 4). Forty-eight (50.0%) of those 96 patients were men and 48 (50.0%) were women, and the median age was 62.5 years (range, 31–87 years). The treatment regimen was based on the cytotoxic agent, hydroxyurea. The initial dose of hydroxyurea was 20–30 mg/kg per day. In case thrombocytosis was not controlled by hydroxyurea, anagrelide HCl was added as 0.5 mg q.i.d. Treatment was continued during the follow-up period through adjusting the dose. All enrolled MPN patients gave their written, informed consent in accordance with the Declaration of Helsinki. This study was approved by the institutional review board of Chonnam National University Hwasun Hospital (Hwasun, Korea).

### Total DNA extraction

Total DNA was extracted from remaining BM specimens, which were collected during BM examinations for diagnostic purposes or as follow–ups and stored in a freezer at −20°C. Commercial kit (QIAamp blood mini kit; Qiagen, Hamburg, Germany) was used for DNA extraction. The concentration of the extracted DNA, measured via spectrophotometry, was 100–400 ng/L.

### Analysis of *JAK2* mutations

#### Quantitative polymerase chain reaction (PCR) for the JAK2 V617F mutation

The *JAK2* V617F mutation was detected via quantitative real-time PCR (*JAK2* MutaQuant assay kit; Ipsogen, Marseille, France) and allele specific-PCR (AS-PCR) [40]. The reaction mixtures contained 5L of the extracted DNA, TaqMan universal PCR master mix (Applied Biosystems; Foster City, CA, USA), primers, TaqMan probe, and distilled water. Real-time PCR was performed using an ABI 7500 system (Applied Biosystems) as follows: 10 minutes at 95°C, 2 minutes at 50°C, 15 seconds at 95°C, and 90 seconds at 63°C; the entire cycle was repeated 50 times. The 5′ end of the TaqMan probe was covalently linked to a reporter dye, either 6-carboxyfluorescein (FAM) or 4,7,2′-trichloro-7′-phenyl-6-carboxyfluorescein (VIC), to monitor its interaction with the *JAK2* V617F mutant allele or the wild-type allele, respectively. Both probes were linked to the quencher dye, tetramethyl-6-carboxyrhodamine at the 3′ end. *JAK2* V617F mutation levels were quantitatively expressed as the FAM/VIC fluorescence ratios in the amplified PCR product. Reactions with positive control (100% *JAK2* V167F) and negative control (0% *JAK2* V167F) were performed along with amplification of the patient's DNA.

#### Direct sequencing for detection of the JAK2 exon 12 mutation

Direct sequencing of exon 12 of the *JAK2* gene was performed in DNA samples from PV patients negative for the *JAK2* V167F mutation [41]. For preliminary amplification of exon 12, the reaction mixtures contained 5μL buffer amplifier (10x), 3μL MgC1_2_ (25mM/L), 2μL dNTPs (containing 2.5 mM of each nucleotide), 1 unit Taq polymerase, 2μL of each primer (10 mM), and 5μL DNA (total volume, 50μL).

The forward primer was 5′-CTCCTCTTTGG AGCAATTCA-3′, and the reverse primer was 5′-GGGAGTTGCGATATAGGTCTT-3′. PCR was performed as follows: 5 minutes at 94°C; 35 cycles of 30 seconds at 94°C, 30 seconds at 50°C, and 30 seconds at 72°C; and 5 minutes at 72°C for extension. The amplification product was verified via electrophoreses on a 1% agarose gel and purified using a PCR purification kit (Qiagen). For direct sequencing, the forward primer was 5′-CTTTGGAGCAATTCATACTTT-3′, and the reverse primer was 5′-AGTTGCGATATAGGTCTTTG-3′. Sequencing kit (Applied Biosystems) and genetic analyzer (ABI 3130XL; Applied Biosystems) were used. The resultant sequences were compared with sequences on the GenBank website (http://www.ncbi.nlm.nih.gov/sites/entrez) using Sequencher v4.1 software (Gene Codes Co., Ann Arbor, MI, USA).

### Detection of *MPL* exon 10 mutations

Direct sequencing of exon 10 of the *MPL* gene was performed in DNA samples from ET and PMF patients negative for the *JAK2* V617F mutation [5]. The reaction mixtures contained 5μL DNA, 10μL 2× PfuPreMix (Biowithus, Seoul, Korea), and 5μL of a mixture of the forward (5′-TGGGCCGAAGTCTGACCCTTT-3′) and reverse (5′-ACAGAGCGAACCAAGAATGCCTGT-3′) primers. *MPL* mutations very frequently occur in codon 515, which is in exon 10. PCR was performed as follows: 5 minutes at 98°C; 35 cycles of 20 seconds at 98°C and 1 minute at 68°C; and 5 minutes at 68°C for extension. The amplification product was verified via electrophoresis on a 1% agarose gel and purified using a PCR purification kit. For direct sequencing, the forward primer was 5′-GGTGACCGCTCTGCATCTAGTGCT-3′, and the reverse primer was 5′-CACCTGGTCCACCGCCAGTCT-3′. The resultant sequences were compared with sequences on the GenBank website using Sequencher v4.1 software.

### Mutation analysis of exon 9 of the *CALR* gene

PCR and DNA fragment analysis, which assesses the size of amplification fragments, were used to detect *CALR* exon 9 mutations. Then, secondary examination using direct sequencing was performed for specimens showing unordinary DNA fragments.

#### DNA fragment analysis for detecting CALR exon 9 mutations

For amplification of exon 9 of the *CALR* gene, the forward primer was 5′-GGCAAGGCCCTGAGGTGT-3′, and the reverse primer was 5′GGCCTCAGTCCAGCCCTG-3′. PCR was performed as follows: 5 minutes at temperatures below 95°C; 10 cycles of 30 seconds at 94°C, 30 seconds at 67°C, and 30 seconds at 72°C (in 1 of the cycles, the annealing temperature was lowered by 1°C); 29 cycles of 30 seconds at 94°C, 30 seconds at 57°C, and 30 seconds at 72°C; and 20 minutes at 72°C for extension. The PCR products were diluted 1:20, and their sizes were measured by using genetic analyzer (ABI 3130XL; Applied Biosystems). Results were analyzed by using GeneMapper software version 4.0 (Applied Biosystems). Direct sequencing was conducted to confirm the *CALR* mutation type.

#### Direct sequencing for confirming the CALR mutation and mutation type

For direct sequencing, the forward primer was 5′-ACAACTTCCTCATCACCAACG-3′, and the reverse primer was 5′-GGCCTCAGTCCAGCCCTG-3′. The resultant sequences were compared with sequences on the GenBank website using Sequencher v4.1 software. *CALR* mutation types were classified as described in previous reports [9, 11, 12].

### Statistical analysis

R version 3.2.4 (The R Foundation for Statistical Computing, Vienna, Austria) was used for the following statistical analyses [38]. The chi-square test or Fisher's exact test was used to analyze differences in non-continuous variables such as sex, splenomegaly, and plateletpheresis. The Kruskal-Wallis test or the Mann-Whitney *U* test was used to analyze continuous variables such as age and hematological index. In 2-sided tests, a *P* value < 0.05 indicated statistical significance. The Cox proportional-hazard regression model was used to analyze the dependency of survival time on predictor variables. Progression-free survival (PFS) was defined as the time from the day of ET diagnosis to the day of progression to PV, post-ET myelofibrosis, and myelodysplastic syndrome or acute leukemia [42]. Partial remission (PR) was defined by the revised response criteria for PV and ET [42]. It includes durable peripheral blood count remission (lasting at least 12 weeks), defined as: platelet count ≤ 400×10^9^/L, WBC count ≤ 10×10^9^/L, absence of leukoerythroblastosis.
